# MicroRNA-326 impairs chemotherapy resistance in non small cell lung cancer by suppressing histone deacetylase SIRT1-mediated HIF1α and elevating VEGFA

**DOI:** 10.1080/21655979.2021.1993718

**Published:** 2022-02-23

**Authors:** Jinying Wei, Guangping Meng, Jing Wu, Ying Wang, Qiang Zhang, Ting Dong, Jin Bao, Chunyan Wang, Jie Zhang

**Affiliations:** a Department of Respiratory and Critical Care Medicine, The Second Hospital of Jilin University, Changchun, P. R. China; b Department of General Practice, The First Hospital of Jilin University, Changchun, P. R. China; c Department of Clinical Laboratory, The First Hospital of Jilin University, Changchun, P. R. China; d Department of Health Examination Center, The First Hospital of Jilin University, Changchun, P. R. China

**Keywords:** Mir-326, sirtuin-1, hif1α, vegfa, non-small cell lung cancer, chemotherapy resistance

## Abstract

Compelling evidence has implicated the role of microRNAs (miRs or miRNAs) in lung cancer. Sirtuin-1 (SIRT1) is a key contributor to the progression of non-small cell lung cancer (NSCLC). This study was intended to investigate whether miR-326 affected NSCLC associated with SIRT1. miR-326 and SIRT1 expression in H460 cells and chemoresistant cells H460-R was measured by RT-qPCR. Dual luciferase reporter gene assay and RIP assay were used to identify and validate the relationship between miR-326 and SIRT1. Using gain- and loss-of-function approaches, we evaluated their effects on the chemoresistance of NSCLC cells. ChIP assay was used to detect binding of SIRT1 to the promoter of HIF1α gene, and the binding H3K9Ac to HIF1α, binding of H3K9Ac and HIF1α after silencing SIRT1, and binding HIF1α to VEGFA promoter. In vivo experiments were performed to validate the *in vitro* findings. MiR-326 expression was decreased while SIRT1 expression was increased in NSCLC cells. SIRT1 was a target of miR-326. MiR-326 inhibited the proliferation of chemotherapy-resistant NSCLC cells and promoted their apoptosis by suppressing SIRT1. In addition, SIRT1 promoted chemoresistance of NSCLC cell by elevating VEGFA expression. Through this mechanism, miR-326 reduced the chemoresistance, which was validated *in vivo*. Taken together, miR-326 represses SIRT1 through impeding HIF1α expression, thus hindering chemotherapy resistance in lung cancer. These findings provide an exquisite therapeutic target for NSCLC.

## Introduction

As the major diagnosed cancer, lung cancer is the main cause of cancer-related deaths and kills nearly 2 million people worldwide each year [1]. Moreover, it was estimated that non-small cell lung cancer (NSCLC) accounts for 85% of lung cancer and effective prevention strategies and potential epigenetic predictive markers are urgently needed due to the limited effects of chemotherapy on NSCLC and the chemotherapy resistance [2,3]. Accumulating researches have identified the potential therapeutic roles of microRNAs (miRNAs) in NSCLC management and complicated mechanisms mediated by these miRNAs [4,5]. Although kinds of diagnostic procedures are available, single-targeted therapies are not the ideal and effective ways in treating and preventing lung cancer, because of drug resistance [6], and biopsy is still essential for routine diagnosis of lung cancer [7]. Therefore, effective prevention strategies and new treatment strategies are needed to prevent, diagnose, and treat lung cancer [8]. Also, it is generally known that developing predictive biomarkers and combining novel drugs that target specific resistance pathways with standard chemotherapy may be some promising strategies for overcoming chemotherapy resistance in lung cancer [9]. Currently, miRNAs might have potential therapeutic use in lung cancer [10].

MiRNAs are important mediators of gene expression that guide their inhibition via pairing to messenger RNAs (mRNAs) of protein-coding genes [11]. Besides, miRNAs can typically inhibit the genes participated in regulation of cellular processes including inflammation, cell cycle progression, stress response, cell differentiation, and apoptosis [12]. Interestingly, dysregulation of miRNAs is implicated in lung cancer development, especially NSCLC [13,14]. For example, miR-326 represses cell proliferation and migration in lung cancer [15] and NSCLC [16], suggesting potential therapeutic treatment for NSCLC, yet the mechanism remains largely unknown. Moreover, miR-326 suppresses progression of lung cancer through a Sp1/KLF3 regulatory axis [17]. Therefore, this study was designed to investigate the mechanism by which miR-326 on the chemotherapy resistance of NSCLC cells and thus to identify a potential targets for NSCLC treatment.

Histone deacetylase is known to be highly conserved in yeast and regulates many proteins involved in chromatin structure, apoptosis, autophagy, and mitochondrial transcriptional regulation [18]. It is reported that SIRT1 serves as a protumorigenic mediator and targeted SIRT1 activity or gene expression may represent a new strategy for lung cancer and NSCLC therapy [3,19,20]. SIRT1 was predicted to be a target of miR-326 prior to our experimentation. Hence, we speculated that the interaction of miR-326 and SIRT1 may function in the pathogenesis of NSCLC. Moreover, overexpression of SIRT1 is a potential therapeutic target for prevention of migration and invasion of lung cancer. Transcription factors and miRNAs have synergistic effects, while have unique molecular mechanisms and evolutionary background, which constitute the two main levels of gene regulatory networks [9]. Transcription factors always act on the binding site of DNA to modulate the expression of genes [21]. HIF1α has been reported to be a direct target of SIRT1 [22]. Also, overexpression of transcription factor HIF1α was detected in cancer cells using ampere immunosensors with nanobiological conjugates [21]. Besides, it is generally known that nicotine enhances cell proliferation in lung cancer through increasing HIF1α [23]. Recently, targeting HIF1α potentiates the efficacy of chemotherapy for colorectal cancer [24]. It was hypothesized that miR-326 and SIRT1 may be involved in chemotherapy resistance of NSCLC *via* mediation of HIF1α.

## Materials and methods

### Ethics statement

The animal experiment was approved by the Experimental Animal Ethics Committee of The Second Hospital of Jilin University and in strict accordance with recommendations in the Guide for the Care and Use of Laboratory Animals of the National Institutes of Health.

### Microarray-based gene expression profiling

MiRNA microarray data related to NSCLC (GSE51853) was retrieved in GEO database, including five normal control samples and 126 NSCLC samples, followed by differential analysis using the Limma package of R language with the threshold of |logFC| > 1, *p* value < 0.05. The possible target genes of microRNA were obtained with the use of StarBase database, RNAInter database, miRanda database. Different algorithms were used, and the intersection of the three prediction results was obtained.

### Cell culture and transfection

Human NSCLC cell line H460 and human embryonic kidney cell line HEK293 were purchased from American Type Culture Collection (ATCC, Manassas, VA, USA). H460 cells were cultured in RPMI-1640, and HEK293 in DMEM in a 37°C, 5% CO_2_ incubator. The chemoresistant cell H460-R was cultured from the parental cell line H460 by exposing to cis-diamminedichloroplatinum II (DDP, cisplatin) from 0.05 μg/mL to a gradient of 2 μg/mL for about 12 months.

### Transfection of plasmids and small interfering RNAs (siRNAs)

MiR-326 mimic, miR-326 inhibitor, overexpressed SIRT1 (oe-SIRT1), small interfering RNA (siRNA) against HIF1α (HIF1α siRNA), vascular endothelial growth factor A (VEGFA) siRNA and their negative controls (NCs) were synthesized by Shanghai GeneChem Co., Ltd. (Shanghai, China). Then, cells were seeded in a 6-well plate overnight, and 2 μg of plasmid was mixed with X-treme GENE HP DNA Transfection Reagent (Roche Applied Science, Basel, Switzerland). Cells were transfected with the above plasmids using Lipofectamine^TM^. Cells were harvested 48 h after transfection.

### RNA isolation and quantitation

Total RNA of tissues or cells was extracted by TRIzol (Invitrogen, Carlsbad, USA). MiRcute Plus miRNA First-Strand cDNA Synthesis Kit (TIANGEN, Beijing, China) was used for reverse transcription of miR-326, and Prime Script^TM^ RT Kit (Takara Biotechnology, Tokyo, Japan) for reverse transcription of SIRT1. TransStart Tip Green qPCR SuperMix (TransGen Biotech, Beijing, China) was used for RT-qPCR experiment. U6 was as internal reference for miR-326 and β-actin for SIRT1. Relative transcription level of target genes was calculated by 2^−ΔΔCt^ method. The primers are shown in Table 1.

**Table 1.** Primer sequences for RT-qPCR

Gene	Primer sequence
miR-326	Forward: 5ʹ- CTCATCTGTCTGTTGGGCTGGAG −3’
	Reverse: 5ʹ- AGGGCCCAGAGGCGATCT −3’
U6	Forward:5ʹ- CTCGCTTCGGCAGCACA −3’
	Reverse:5ʹ- AACGCTTCACGAATTTGCGT −3’
SIRT1	Forward: 5ʹ- CTTTGCCTCATCTGCATTTT −3’
	Reverse: 5ʹ- ATTAGGCCAGCATTTTCTCA −3’
β-actin	Forward: 5ʹ- ACCAACTGGGACGACATGGAG-3’
	Reverse: 5ʹ- GTGAGGATCTTCATGAGGTAGTC −3’

Note: RT-qPCR, reverse transcription quantitative polymerase chain reaction; MiR-326, microRNA-326; SIRT1, Sirtuin-1; β-actin, beta-actin

### Western blot analysis

The cells were suspended in RIPA buffer to isolate total protein. The protein was separated by SDS-PAGE and then transferred to polyvinylidene fluoride membrane. The membrane was incubated with primary antibody overnight at 4°C, incubated with specific secondary antibody, developed by enhanced chemiluminescence, and exposed in the Amersham Imager 600 system (GE Healthcare Life Sciences, Shanghai, China). The used primary antibodies were SIRT1 (ab32441, 1: 20,000), HIF1α (ab51608, 1: 100), VEGFA (ab52917, 1: 10,000), c-PARP1 (ab32064, 1: 1000), c-Caspase-3 (ab13847, 1: 500), Ɣ-H2AX (ab2893, 1: 1000), β-Actin (ab179467, 1: 5000, all from Abcam Inc., Cambridge, UK), and secondary antibody used were immunoglobulin G (IgG) (ab6721, 1: 2000, Abcam Inc.).

### Cell viability assay

H460-R cells were seeded in a 96-well plate at 7000 cells/well for 12–16 h. Next, cells were treated with cis-diamminedichloroplatinum (DDP) at 0, 2, 4, 8, and 16 μg/mL for 72 h, respectively. Cell viability was determined using the 3-(4,5-dimethylthiazol-2-yl)-5-(3-carboxymethoxyphenyl)-2-(4-sulfophenyl)-2 H-tetrazolium, inner salt (MTS) kit (Abcam Inc.).

### Flow cytometry assay

After treating the cells with DDP at 4 μg/mL for 72 h, the trypsin-digested cells were thoroughly mixed in 1 × binding buffer. Then, cell suspension was added with 5 μL of Annexin V and 5 μL of propidium iodide (PI) and incubated for 15 min. Apoptotic cells were assayed using the FITC Annexin V Apoptosis Detection Kit I (BD Bioscience, MA, USA).

### Immunofluorescence assay

The cells treated with DDP at 4 μg/mL were treated with 4% paraformaldehyde for 15 min, permeabilized with 0.2% Triton-X100 for 5 min, and blocked with PBS buffer containing 5% BSA for 1 h. After that, cells were incubated overnight at 4°C with murine monoclonal anti-γ-H2AX antibody (ab195189, 1: 200, Abcam Inc.), and incubated for 1 h with Alexa Fluor 488 conjugated secondary antibody. Then, cells were incubated with DAPI buffer for 5 min, and observed and analyzed using LSM 710 confocal microscopy.

### Dual luciferase reporter gene assay

The wild type (WT) or mutant (MUT) sequence at the 3ʹUTR of SIRT1 was synthesized and cloned into pMIR-reporter. The vector of SIRT1 WT 3ʹUTR or MUT 3ʹUTR was co-transfected with miR-326 mimic or NC mimic into HEK293T cells using Lipofectamine 2000. After 48 h of transfection, cells were harvested to detect luciferase activity.

The UCSC and JASPAR websites were used to analyze the possible binding sites of HIF1α protein in the VEGFA promoter. And truncation or recombinant luciferase reporter vector with mutation binding site was co-transfected into HEK293T cells with HIF1α expression vector. Oe-NC and oe-HIF1α were co-transfected with the VEGFA promoter region 2Kb luciferase reporter plasmids to detect whether HIF1α could bind to the VEGFA promoter region. Renilla luciferase expression vector pRL-TK (TaKaRa, Dalian, China) was used as an internal reference. After 48 h of transfection, cells were harvested and lysed. Luciferase reporter assays were performed using the dual luciferase reporter assay system.

### RIP

EZ-Magna RIP kit (Millipore) was used for RIP experiment. Each group of cells was cultured with NP-40 RIP lysis buffer containing dithiothreitol (DTT) (1 mM), phenylmethanesulfonyl fluoride (PMSF) (1 mM), RNase inhibitor (200 U/mL) and 1% protease inhibitor. RIP buffer with magnetic beads conjugated by AGO2 antibody (ab186733, 1: 30, Abcam Inc.) was added to whole-cell lysate (200 μL) with IgG antibody (ab6720, 1: 50, Abcam Inc.) as a NC. The beads were washed with pre-cooled NT2 buffer and incubated with proteinase K (10 mg/mL) for 30 min. All immunoprecipitated RNA interacting with AGO2 was harvested by TRIzol reagent and detected by RT-qPCR.

### ChIP

Enrichment of HIF1α in the promoter region of VEGFA was determined using the ChIP kit (Millipore). Cells were added 1% formaldehyde and fixed for 10 min to cross-link the DNA and protein. After cross-linking, cells were randomly broken by ultrasonic treatment (10 s/time, interval 10 s, cycle 15 times) into fragments. The cells were centrifuged at 4°C, and the supernatant was collected. Then, NC IgG antibody (ab6721, 1: 30, Abcam Inc.) and the target protein-specific antibody HIF1α (ab2185, 1: 1000, Abcam Inc.) were added, respectively, and cells were incubated overnight at 4°C. Protein Agarose/Sepharose was used to precipitate endogenous DNA-protein complexes. After centrifugation, nonspecific complex was washed off. Cells were de-crosslinked at 65°C overnight, and DNA fragment was purified by phenol/chloroform extraction to examine binding of HIF1α to the VEGFA promoter region.

### Tumor formation in nude mice

H460 cells that were transfected with miR-326 agomir or miR-326 NC were suspended in 0.2 mL PBS and Matrigel mixture at 5 × 10^6^ and subcutaneously injected into BALB/C nu/nu nude mice (female, 4–6 weeks, n = 8/group, Sun Yat-sen University, Guangdong, China). All mice were kept in conditions without pathogens. One week later, mice were treated with DDP (weighing 3.0 mg/kg) every 3 d, and tumor volume was detected for 4 weeks. And the tumor volume was measured every 3 or 4 d, and the formed tumor volume was calculated. The mice were euthanized by cervical dislocation, and then tumors were harvested and weighed.

### Statistical analysis

Unpaired data in compliance with normal distribution and homogeneity between two groups were compared using unpaired *t*-test. Comparisons among multiple groups at different time points were conducted by repeated measurement one-way ANOVA, followed by a Bonferroni’s post hoc test for multiple comparisons, while other comparisons among multiple groups were conducted by one-way ANOVA with Bonferroni’s post-hoc test. A value of *p* < 0.05 indicates significant difference. The experiment was independently repeated three times.

## Results

### MiR-326 is poorly expressed while SIRT1 is highly expressed in lung cancer

**Figure 1.**
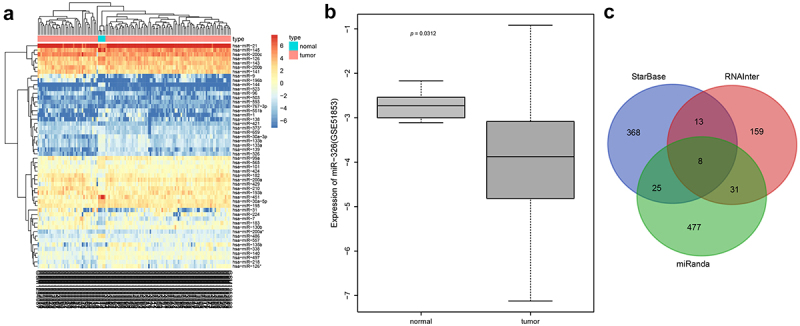
Differential analysis and biosignal prediction of NSCLC miRNA-related microarray GSE51853. A, Differential miRNAs expression heat map, abscissa indicated sample and ordinate indicated differential miRNAs. B, Expression of miR-326 in NSCLC sample and normal control sample in microarray GSE51853. C, The Venn diagram of StarBase database, RNAInter database and miRanda database for intersection of prediction results of miR-326 targeted genes.

Differential analysis of NSCLC miRNA-related expression microarray GSE51853 was performed, and 52 differential miRNAs were obtained, and then a heatmap of expression of differential miRNAs was plotted (Figure 1(a)). Among them, miR-326 was poorly expressed in NSCLC (Figure 1(b)), and miR-326 inhibited the chemotherapy resistance of lung adenocarcinoma cells [25]. StarBase database, RNAInter database and miRanda database were used to predict the target genes of miRNA, and eight target genes (RUNX3, MAGI3, SH3PXD2A, BSDC1, SRRM1, CELF2, RIT1, SIRT1) were obtained (Figure 1(c)). Among them, SIRT1 was highly expressed in chemotherapy-resistant lung cancer cells and promoted chemotherapy resistance of lung cancer cells [26]. So SIRT1 was selected for the next experiments.

### MiR-326 inhibits SIRT1 expression

**Figure 2.**
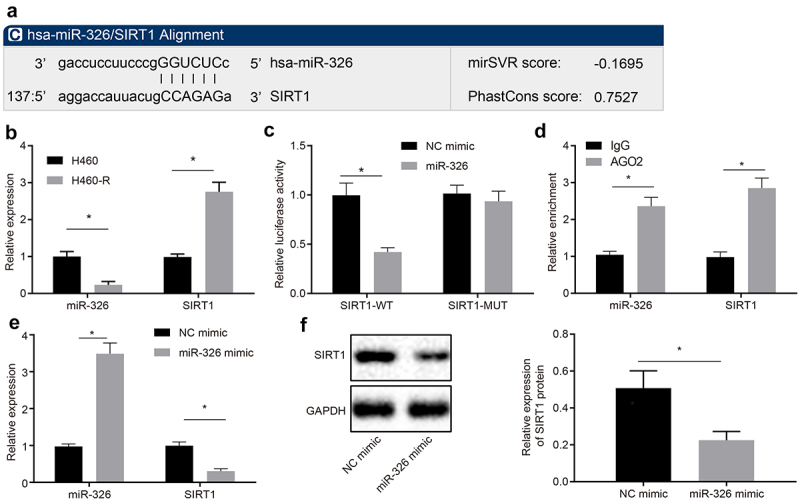
MiR-326 targets SIRT1. A, The website predicted that miR-326 targeted the 3ʹUTR region binding site of SIRT1. B, Detection of miR-326 and SIRT1 expression in H460 cells and chemoresistant cells H460-R by RT-qPCR. C, Verification of relationship between miR-326 and SIRT1 by dual luciferase reporter gene assay. D, Detection the combination of miR-326 and SIRT1 by RIP assay. E, Detection of miR-326 and SIRT1 expression by RT-qPCR. F, Analysis of SIRT1 expression in each group of cells by Western blot analysis. Measurement data between two groups were compared using paired *t*-test. The experiment was independently repeated three times. * *p* < 0.05 *vs*. H460 cells or cells transfected with NC mimic or treated by IgG.

Website (microRNA.org) predicted that miR-326 could target the 3ʹUTR region of the histone deacetylase SIRT1 (Figure 2(a)), but the interaction between the two has not been reported. Therefore, RT-qPCR was conducted to measure miR-326 and SIRT1 expression in H460 cells and chemoresistant cells H460-R, and results showed that compared with H460 cells, miR-326 expression in H460-R was downregulated, and SIRT1 expression was upregulated (all *p* < 0.05) (Figure 2(b)).

To verify the relationship between miR-326 and SIRT1, a SIRT1 MUT was constructed. Results showed that miR-326 inhibited luciferase activity of SIRT1-WT, but has no effect on luciferase activity of SIRT1-MUT (*p* < 0.05) (Figure 2(c)). The binding of miR-326 to SIRT1 was detected by RIP. It was revealed that AGO2 antibody-enriched miR-326 and SIRT1 were increased (Figure 2(d)), indicating that miR-326 and SIRT1 bound to each other. Expression of miR-326 and SIRT1 was detected by RT-qPCR and Western blot analysis, and it was demonstrated that compared with cells transfected with NC mimic, miR-326 expression in cells transfected with miR-326 mimic was upregulated, and expression of SIRT1 was downregulated (all *p* < 0.05) (Figure 2(e -f)). Taken together, miR-326 can repress the expression of SIRT1 by binding to its 3ʹUTR region.

### MiR-326 inhibited chemoresistance of NSCLC cells by repressing SIRT1

**Figure 3.**
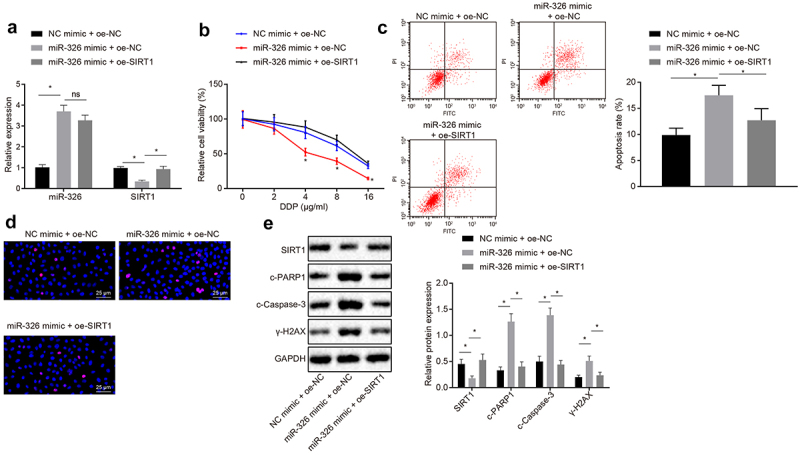
MiR-326 suppresses chemoresistance of NSCLC cells by negatively regulating SIRT1. A, Measurement of miR-326 and SIRT1 expression by RT-qPCR. B, MTS assay for proliferation of H460-R cells treated with different concentrations of DDP. C, Flow cytometry assay for apoptosis of H460-R cells treated with 4 μg/mL DDP. D, Immunofluorescence assay detection of Ɣ-H2AX positive expression in cells (100 ×). E, Protein level of SIRT1, c-PARP1, c-Caspase-3 and Ɣ-H2AX in H460-R cells after DDP treatment by Western blot analysis. The experiment was independently repeated three times. * *p* < 0.05 *vs*. upon treatment of miR-326 mimic and oe-NC.

To confirm the regulation of miR-326 targeting SIRT1 in chemotherapy resistance of NSCLC cells, chemotherapy resistant H460-R cells were co-transfected with NC mimic and oe-NC, miR-326 mimic and oe-NC, or miR-326 mimic and oe-SIRT1. miR-326 and SIRT1 expression was detected by RT-qPCR and results demonstrated that miR-326 expression was raised and SIRT1 expression was decreased in cells treated with miR-326 mimic and oe-NC compared with NC mimic and oe-NC treatment. In relation to miR-326 mimic and oe-NC treatment, miR-326 expression had no significant difference and SIRT1 expression was enhanced in cells treated with miR-326 mimic and oe-SIRT1 (all *p* < 0.05) (Figure 3(a)).

The survival rate of H460-R cells treated with different concentrations of DDP was determined by MTS method and apoptosis of H460-R cells treated with 4 μg/mL DDP was detected by flow cytometry assay. According to the results, it was signified that in contrast with NC mimic and oe-NC treatment, the cell proliferation rate was decreased, and apoptosis rate was increased after miR-326 mimic and oe-NC treatment. Compared with miR-326 mimic and oe-NC treatment, cell proliferation rate was elevated and apoptotic rate was increased after miR-326 mimic and oe-SIRT1 treatment (*p* < 0.05) (Figure 3(b -c)).

Immunofluorescence assay was used to detect Ɣ-H2AX positive expression in cells to detect DNA damage, and results revealed that positive expression of Ɣ-H2AX was green fluorescence, and the positive expression of Ɣ-H2AX was increased in miR-326 mimic and oe-NC treatment compared with NC mimic and oe-NC treatment. Ɣ-H2AX expression was reduced in cells transfected with miR-326 mimic in contrast with cells transfected with miR-326 mimic and oe-SIRT1 (Figure 3(d)).

The protein levels of SIRT1, c-PARP1, c-Caspase-3 and Ɣ-H2AX in H460-R cells after DDP treatment were analyzed by Western blot analysis and results demonstrated that compared with cells treated with NC mimic and oe-NC, SIRT1 protein expression was downregulated, and protein expression of c-PARP1, c-Caspase-3, and Ɣ-H2AX was upregulated in cells treated with miR-326 mimic and oe-NC. In response to upon treatment of miR-326 mimic and oe-NC, SIRT1 protein expression was raised and oe-SIRT1, and protein expression of c-PARP1, c-Caspase-3 and Ɣ-H2AX was reduced in upon treatment of miR-326 mimic (all *p* < 0.05) (Figure 3(e)). To conclude, miR-326 could negatively regulate the expression of SIRT1 to inhibit the chemotherapy resistance of NSCLC cells.

### Silencing of SIRT1 expression promotes degradation of transcription factor HIF1α to inhibit chemoresistance of NSCLC cells

**Figure 4.**
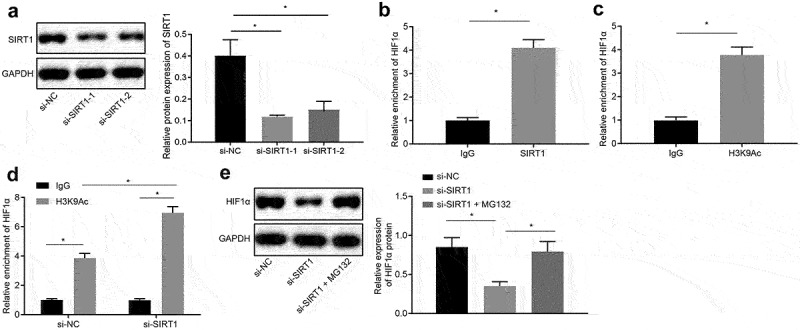
Silencing the expression of SIRT1 promotes the degradation of the transcription factor HIF1α. A, Analysis the silencing efficiency of SIRT1 by Western blot analysis. B, Detection of SIRT1 binding to HIF1 by ChIP assay. C, Detection of H3K9Ac binding to HIF1 by ChIP assay. D, Detection of H3K9Ac binding to HIF1 after silencing SIRT1 by ChIP assay. E, Analysis of HIF1α expression in si-NC-treated cells and si-SIRT1-treated cells. The experiment was independently repeated three times. * *p* < 0.05 *vs*. upon treatment of si-NC.

Next, the effect of SIRT1 on the chemotherapy resistance of NSCLC through the downstream gene HIF1α was investigated. The silencing efficiency of SIRT1 was analyzed by Western blot analysis and it was revealed that compared with si-NC-treated cells, SIRT1 expression was decreased in si-SIRT1-1-treated cells and si-SIRT1-2-treated cells (*p* < 0.05) (Figure 4(a)), and the si-SIRT1-1-treated cells sequence was selected as the silencing SIRT1 for subsequent experiments. ChIP assay demonstrated that SIRT1 was recruited in the HIF1α promoter region, and H3K9Ac was enriched in the HIF1α promoter region (Figure 4(b-c)). Then, after silencing SIRT1, the H3K9Ac recruited in the HIF1α promoter region was increased in si-SIRT1-treated cells compared with si-NC-treated cells (Figure 4(d)).

H460-R cells were treated with 10 μM proteasome inhibitor MG132 (Sigma Aldrich, St. Louis, MO, USA) [27], and Western blot analysis was used to analyze expression of HIF1α in si-NC-treated cells and si-SIRT1 treated cells. The results proved that in contrast with cells transfected with si-NC, the expression of HIF1α in cells transfected with si-SIRT1 was downregulated, and in presence of the proteasome inhibitor MG132, the inhibitory effect of si-SIRT1 treatment on HIF1α was alleviated (*p* < 0.05) (Figure 4(e)), indicating that silencing SIRT1 promoted proteasomal degradation of HIF1α by inhibiting its deacetylation.

**Figure 5.**
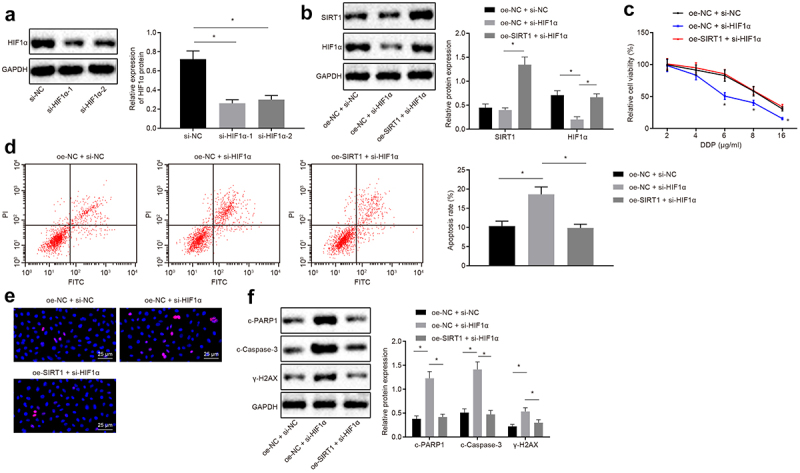
Silencing SIRT1 promotes the degradation of HIF1α to inhibit chemotherapy resistance in NSCLC cells. A, Analysis of HIF1α screening silenced sequence by Western blot analysis. B, Analysis of HIF1α and SIRT1 expression by Western blot analysis. C, MTS assay for proliferation of H460-R cells treated with different concentrations of DDP. D, Flow cytometry assay for detection of apoptosis in H460-R cells. E, Detection of Ɣ-H2AX positive expression in cells by immunofluorescence assay. F, Analysis of protein levels of SIRT1, c-PARP1, c-Caspase-3 and Ɣ-H2AX in H460-R cells by Western blot analysis. The experiment was independently repeated three times. * *p* < 0.05 *vs*. upon treatment of si-NC or oe-NC + si-NC.

Western blot analysis uncovered that si-HIF1α-1 treatment with better silencing efficiency was selected for subsequent experiments (Figure 5(a)). H460-R cells were then co-transfected with oe-NC and si-NC, oe-NC and si-HIF1α, oe-SIRT1 and si-HIF1α1. It was observed that in response to cells co-transfected with oe-NC and si-NC, there was no significant difference in SIRT1 protein expression, and HIF1α was decreased in cells co-transfected with oe-NC and si-HIF1α. Contrast to cells co-transfected with oe-NC and si-HIF1α, SIRT1 protein expression was elevated in cells co-transfected with oe-SIRT1 and si-HIF1α (Figure 5b).

MTS assay and flow cytometry assay revealed that compared with cells co-transfected with oe-NC and si-NC, the cell proliferation was decreased, and apoptosis rate was increased in cells co-transfected with oe-NC and si-HIF1α. Compared with cells co-transfected with oe-NC and si-HIF1α, the proliferation rate was increased, and apoptosis rate was reduced in cells co-transfected with oe-SIRT1 and si-HIF1α (*p* < 0.05) (Figure 5(c -d)). Immunofluorescence and Western blot analysis illustrated that protein expression of c-PARP1, c-Caspase-3, and Ɣ-H2AX was upregulated in cells treated with oe-NC and si-HIF1α compared with cells treated with oe-NC and si-NC, while compared with cells treated with oe-NC and si-HIF1α, the protein expression of c-PARP1, c-Caspase-3, and Ɣ-H2AX positive expression was downregulated in cells treated with oe-SIRT1 and si-HIF1α (all *p* < 0.05) (Figure 5(e -f)).

In short, SIRT1 silencing could promote the degradation of HIF1 and hence inhibit the chemotherapy resistance of NSCLC cells.

### SIRT1 raises VEGFA expression by inhibiting HIF1α expression

**Figure 6.**
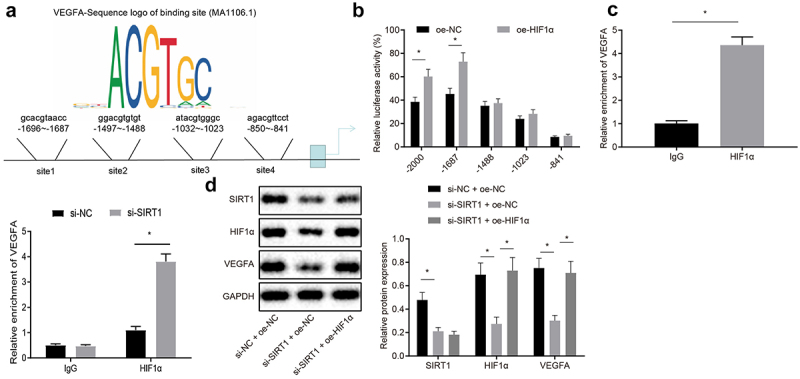
SIRT1 elevates VEGFA expression via repressing HIF1α. A, Prediction of possible binding sites for HIF1α and VEGFA promoters by JASPAR website; B, Verification of binding relation between HIF1α and VEGFA promoters by dual luciferase reporter gene assay. C, Detection binding condition of HIF1α binding to promoter 2 of VEGFA by ChIP assay. D, Analysis of VEGFA expression by Western blot analysis. The experiment was independently repeated three times. * *p* < 0.05 *vs*. upon treatment of upon treatment of IgG, oe-NC, upon treatment of si-NC + oe-NC.

JASPAR website was used to predict possible binding sites for HIF1α and VEGFA promoters (Figure 6(a)). Dual luciferase reporter gene assay was used to verify the predicted results and it was presented that HIF1α and VEGFA was bound at site 2 on the promoter (*p* < 0.05) (Figure 6(b)). ChIP assay proved that HIF1α was recruited at promoter region 2 of VEGFA, and the binding of HIF1α and VEGFA site 2 was reduced after silencing SIRT1 (*p* < 0.05) (Figure 6(c)). Western blot analysis was used to analyze VEGFA expression, and it was revealed that compared with upon treatment of si-NC and oe-NC, the expression of VEGFA protein upon treatment of si-SIRT1 and oe-NC was reduced, while elevated upon treatment of si-SIRT1 and oe-HIF1α in contrast to si-SIRT1 and oe-NC treatment (*p* < 0.05) (Figure 6(d)). To sum up, VEGFA expression was elevated by SIRT1 through HIF1α repression.

### SIRT1 promotes chemotherapy resistance of NSCLC by increasing expression of VEGFA

**Figure 7.**
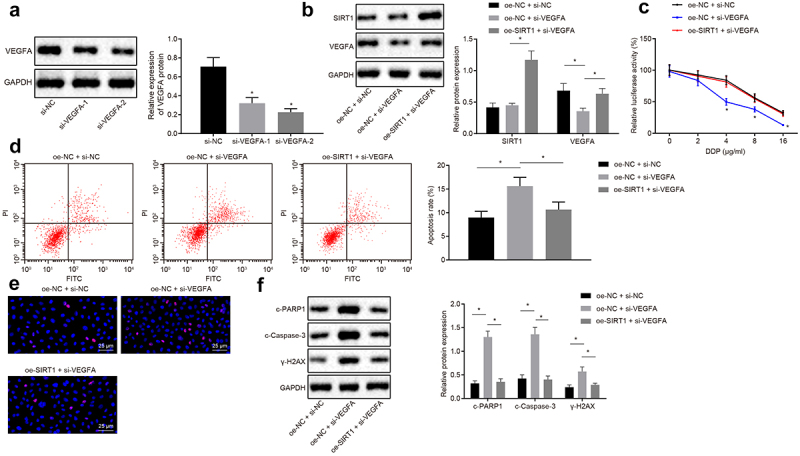
SIRT1 facilitates chemotherapy resistance of NSCLC by boosting VEGFA expression. A, Western blot analysis of VEGFA screening silenced sequence. B, Analysis of VEGFA and SIRT1 expression by Western blot analysis. C, MTS assay for proliferation of H460-R cells. D, Flow cytometry assay for detection of apoptosis in H460-R cells. E, Detection of Ɣ-H2AX positive expression in cells by immunofluorescence assay. F, Analysis of protein levels of SIRT1, c-PARP1, c-Caspase-3 and Ɣ-H2AX in H460-R cells by Western blot analysis. The experiment was independently repeated three times. * *p* < 0.05 *vs*. si-NC treatment or oe-NC + si-NC treatment.

Western blot analysis was used to analyze VEGFA screening silenced sequence, and si-VEGFA-2 treatment with better silencing efficiency was selected for subsequent experiments (Figure 7(a)). Western blot analysis was used to analyze VEGFA and SIRT1 expression after the treatment of oe-NC + si-NC, oe-NC + si-VEGFA, or oe-SIRT1 + si-VEGFA., and it was proved that compared with oe-NC and si-NC treatment, VEGFA expression was downregulated in oe-NC and si-VEGFA treatment. Compared with oe-NC and si-VEGFA treatment, expression of SIRT1 and VEGFA protein was upregulated in the oe-SIRT1 and si-VEGFA treatment (*p* < 0.05) (Figure 7(b)).

MTS and flow cytometry assay revealed that in contrast with cells co-transfected with oe-NC and si-NC, the cell proliferation rate was decreased, and apoptosis rate was increased in cells co-transfected with oe-NC and si-VEGFA. Compared with cells co-transfected with oe-NC and si-VEGFA, the proliferation rate was enhanced, and apoptosis rate was decreased in cells co-transfected with oe-SIRT1 and si-VEGFA (*p* < 0.05) (Figure 7(c -d)).

Immunofluorescence and Western blot analysis demonstrated that protein expression of c-PARP1, c-Caspase-3, and Ɣ-H2AX was accelerated in cells treated with oe-NC and si-VEGFA compared with cells treated with oe-NC and si-NC, while compared with cells treated with oe-NC and si-VEGFA, the protein expression of c-PARP1, c-Caspase-3, and Ɣ-H2AX was decelerated in cells treated with oe-SIRT1 and si-VEGFA (all *p* < 0.05) (Figure 7(e)). In a word, chemotherapy resistance of NSCLC cells was enhanced by SIRT1-upregulated VEGFA.

### Overexpression of miR-326 inhibits chemotherapy resistance of NSCLC cells in vivo

**Figure 8.**
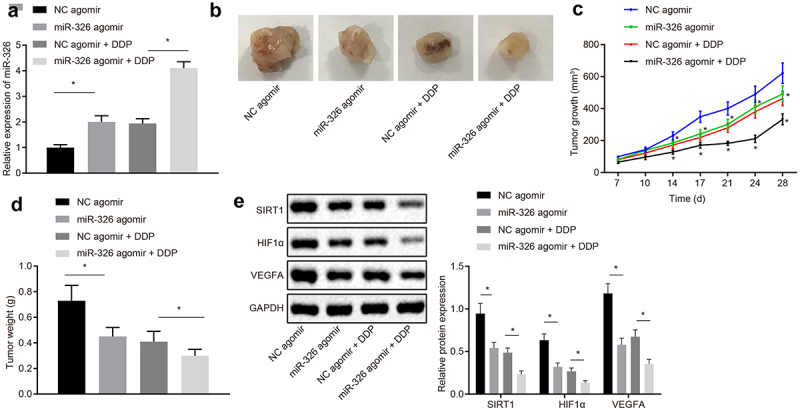
Overexpression of miR-326 represses chemotherapy resistance of NSCLC cells *in vivo*. A, Measurement of miR-326 expression by RT-qPCR. B, Representative images of tumors. C,Tumor growth curve of mice. D, Tumor weight of mice. E, Analysis of SIRT1, HIF1α and VEGFA expression by Western blot analysis. The experiment was independently repeated three times. * *p* < 0.05 *vs*. mice injected with NC agomir, mice injected with NC agomir and DDP, mice injected with NC agomir, mice injected with NC agomir.

RT-qPCR was employed to measure the expression of miR-326 in mice after different treatments, and it was revealed that in contrast with mice injected with NC agomir, miR-326 expression was upregulated in mice injected with miR-326 agomir. And miR-326 expression was raised in mice injected with miR-326 agomir and DDP compared with the mice injected with NC agomir and DDP (all *p* < 0.05) (Figure 8(a)).

The tumor growth curve and weight of each group of mice was detected and results proved that tumor volume and weight was declined in mice injected with miR-326 agomir compared to mice injected with NC agomir. Tumor volume and weight of DDP-treated mice was reduced in mice injected with miR-326 mimic (all *p* < 0.05) (Figure 8(b -d)).

SIRT1, HIF1α, and VEGFA expression was analyzed by Western blot analysis and results shown that expression of SIRT1, HIF1α, and VEGFA protein was declined in mice injected with miR-326 agomir compared with mice injected with NC agomir. SIRT1, HIF1α and VEGFA protein expression in DDP-induced chemotherapy-resistant lung cancer tissues were declined when miR-326 was overexpressed (Figure 8(e)). It was concluded that chemotherapy resistance of NSCLC cells was suppressed by miR-326 overexpression *in vivo*.

## Discussion

**Figure 9.**
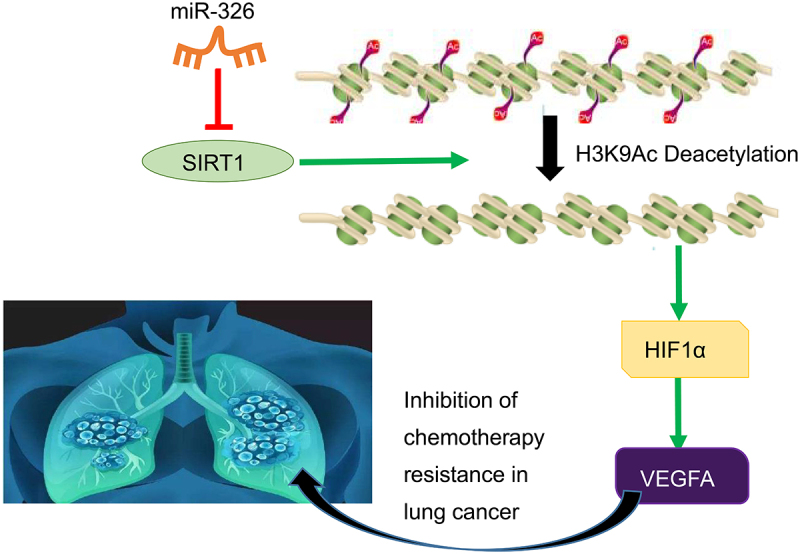
The molecular mechanism graph of the regulatory network and function of SIRT1. MiR-326 downregulates transcription factor HIF1α to upregulate VEGFA *via* blocking histone deacetylase SIRT1, whereby inhibiting chemotherapy resistance in NSCLC.

Lung cancer is the major reason of cancer death worldwide, leading to the same deaths of the combination of four deadliest cancers including breast, prostate, colon, and pancreatic [28]. It is reported that among all lung cancer cases, NSCLC accounts for 85% [2]. Treatments for NSCLC include surgery, radiation, chemotherapy, and systemic therapy according to different stages, and the tendency of future management will involve in different treatment modalities and is highly dependent on histology and biomarkers [29]. MiRNAs are crucial gene regulator in normal cells and lung cancer cells, and anticancer effect in the tumorigenesis of lung cancer [30]. One set of signature miRNAs may be promising biomarker for early screening of high-risk populations and early diagnosis of lung cancer [31]. In addition, our study further evidenced that miR-326 inhibited chemotherapy resistance of NSCLC by downregulating SIRT1 and HIF1α expression and upregulating VEGFA expression (Figure 9).

In the subsequent experiments, miR-326 was expressed at low level in NSCLC cells, and miR-326 inhibited chemotherapy resistance of lung cancer cells. MiR-326 is poorly expressed in lung cancer [15]. Besides, miR-326 was found to be very low level in NSCLC cell lines [32]. More importantly, miR-326 promotes the progression of NSCLC [33]. It is verified that miR-326 reverses chemoresistance in human lung adenocarcinoma cells. While our study validated that SIRT1 [34] was highly expressed in NSCLC cells and it promoted chemotherapy resistance of NSCLC cells. It is verified that SIRT1 is expressed at high level in brain metastasis tissues of NSCLC [35]. Histone deacetylases are an important family of 18 isozymes, and one of the most studied epigenetic regulators in various cancer. Expression of SIRT1 [36] has been proved in a prior study to be related to NSCLC progression [37]. Collectively, miR-326 suppressed chemotherapy resistance of NSCLC cells by inhibition of SIRT1.

MiR-326 inhibited SIRT1 to repress the proliferation of NSCLC cells, thereby promoting its apoptosis and reducing the degree of DNA damage. MiR-30a suppresses lung cancer progression by targeting SIRT1 [38]. It is confirmed that miR-326 promoted apoptosis and phorbol myristate acetate-induced differentiation [39]. Besides, miR-326 reduces the growth of hepatocellular carcinoma cells *via* promoting apoptosis [40]. Those findings were partially consistent with our earlier observations that miR-326 contributed to apoptosis in NSCLC cells. Additionally, as demonstrated in a previous study that apoptosis-related molecules such as c-PARP1 might be the primary independent adverse prognostic factor in lung cancer [41]. Besides, when apoptosis signaling occurs, cleaved (c-) caspase-3 leads to degradation of neurons membrane to prevent repair of damaged DNA [42]. Additionally, apoptosis was characterized by regulation of markers such as increase of c-caspase 3, and c-PARP proteins [43]. Phosphorylated histone H2AX (Ɣ-H2AX), a central player in DNA damage response, is a marker of DNA damage *in vitro* and *in vivo* [44]. In addition, Ɣ-H2AX regulates apoptosis in lung cancer cells [45]. miR-326 induced apoptosis, which was shown in conjunction with the upregulation of key apoptosis protein c-caspase-3 [32]. It is verified that SIRT1 inhibited apoptosis by down-regulating the pro-apoptotic markers, such as cleaved caspase-3 and cleaved PARP [46]. Our study suggested that miR-326 inhibited SIRT1 to repress the chemoresistance of NSCLC cells *via* targeting SIRT1.

This present study further confirmed that SIRT1 increased VEGFA expression by inhibiting HIF1α in order to enhance chemotherapy resistance of NSCLC cells. Transcription factors are reported to play an important role in carcinogenesis and are therefore becoming increasingly popular as potential therapeutic targets in drug growth [47]. A prior study identified HIF1α as a key mediator in hypoxia-induced NSCLC cell proliferation [48]. The overexpression of wild-type SIRT1 enhanced the accumulation of HIF1α [49]. Besides, HIF1α enhances cisplatin resistance in lung cancer by regulating Xeroderma pigmentosum complementation group A expression [50]. SRC-1 promotes tumor angiogenesis by raising HIF1α-mediated VEGFA expression [51]. In addition, VEGFA activates the erythropoietin receptor and contributes to pathologic angiogenesis mediated by VEGFR2 [52]. VEGFA regulates lung cancer migration and invasion by PI3K/AKT pathway [53]. Furthermore, it is consistent found that down-regulation of VEGFA by miR-29 c can impair the proliferation of NSCLC cells [54]. On the aforementioned, it was concluded that SIRT1 elevated VEGFA expression and consequently promoted NSCLC growth by diminished the suppression of HIF1α.

## Conclusions

In conclusion, miR-326 inhibited SIRT1 expression to repress HIF1α and elevate VEGFA in order to reduce chemotherapy resistance, thereby suppressing the development of NSCLC. Thus, miR-326 might serve as a promising therapeutic strategy for NSCLC. However, there still are deficiencies in our study. Our study has not been verified in clinical trials so that we cannot guarantee whether our experimental results are consistent in clinical trials. Therefore, further experiments are needed to ensure the accuracy of our research results.

## Data Availability

The data and materials of the study can be obtained from the corresponding author upon request.
